# Divided zygoma in Holocene human populations from Northern China

**DOI:** 10.1002/ajhb.23314

**Published:** 2019-08-28

**Authors:** Qun Zhang, Quanchao Zhang, Shiyu Yang, Paul C. Dechow, Hong Zhu, Hui‐Yuan Yeh, Qian Wang

**Affiliations:** ^1^ School of Humanities Nanyang Technological University Singapore Singapore; ^2^ School of Archaeology Jilin University Jilin China; ^3^ Department of Biomedical Sciences Texas A&M University College of Dentistry Dallas Texas

## Abstract

**Objectives:**

Divided zygoma (DZ) occurs in contemporaneous human populations, with the highest incidences in people from East Asia and Southern Africa. The present study examines the prevalence and variation of this condition in the Holocene populations of Northern China for the first time.

**Methods:**

In this study, 1145 skulls from various human populations living in Northern China from the Neolithic Age to recent dynasties (5000‐300 years BP) were examined. Specifically, cranial measurements and a CT scan were conducted to quantify craniofacial morphology.

**Results:**

Fifteen skulls were identified with DZ, revealing an overall prevalence of 1.3% in the collection, while it was determined to be higher in North Asian and Northeast Asian regional groups. In skulls with unilateral DZ, the superior division of the zygoma was generally slender, while the inferior division of the zygoma was more robust. In skulls with bilateral DZ, the maxillae were generally more laterally extended. Moreover, unilateral DZ skulls displayed differences in cortical bone thickness between two sides of the facial skeleton.

**Discussion:**

In context, the distribution pattern within these data points toward a greater prevalence of the DZ phenotype in North and Northeast Asian regional groups, suggesting a hypothesis that the DZ trait is more frequent in populations characterized by flat and broad faces. Accordingly, further studies into the DZ condition will deepen our understanding of developments in plasticity, variation, and recent evolution of the human cranium.

## 1 INTRODUCTION

The zygomatic bones are an important component of the midfacial skeleton, and they play a critical role in the support and integration of the craniofacial skeleton as well as the masticatory apparatus. Each zygoma is normally quadrangular in morphology and articulates with the ipsilateral maxilla, frontal, temporal, and sphenoidal bones. Functionally, the zygoma exists as a crucial force dispersion and kinetic energy absorption site in the midface while being situated in the lateral zygomatico‐maxillary buttress (Hardt & Kuttenberger, 2010). Furthermore, each zygoma provides an attachment for a masseter muscle, and plays an important functional role in the masticatory system. Thus, the integrity of zygomatic bones is very important in maintaining normal function of the facial skeleton (Wang & Dechow, 2016). Apart from its practical applications, the zygoma has multiple variations among different populations due to differences in genetic and environmental influences, including population history. The bone can change in shape and size (Noback & Harvati, 2015; Oettlé, Demeter, & L'abbé, 2017; Oschinsky, 1962; Paschetta et al., 2010), presenting morphological differences in zygomatic projection, zygomaxillary tuberosity, the malar tubercle, zygomatic trigone, and the course of the zygomaxillary suture (Baab et al., 2010; Hefner, 2009; İşcan & Steyn, 2013; L'Abbé et al., 2011; Lahr & Wright, 1996; Oettlé et al., 2017; Oschinsky, 1962; Standring, 2008; Vitek, 2012). Yet, in a natural although rare form, the zygoma can also be divided by extra sutures into two or more parts (Hanihara, Ishida, & Dodo, 1998; Hrdlička, 1902; Ossenberg, 2013; Wang & Dechow, 2016). To illustrate, in most divided zygoma (DZ) phenotypes, the bone is transected by a supernumerary suture that divides the facial aspect of the zygoma into upper and lower divisions, giving the bone a bipartite morphology termed *os zygomaticum bipartitum*, previously called *os japonicum* due to its high prevalence in the Japanese population (Hrdlička, 1902).

In the past century, the condition of DZ was investigated among several populations, specifically in the Native Americans (Hrdlička, 1902, 1904; Oetteking, 1930), Asians (Anil, Peker, Turguta, Pelin, & Gülekonc, 2000; Bhargava, Garg, & Bhargava, 1960; Jeyasingh, Gupta, Arora, & Saxena, 1982; Koganei, 1926; Kundu et al., 2016; Mangalgiri, Satpathy, & Bhojwani, 2015; Ohnishi, 1940; Soni & Khatri, 2016), Europeans (Dimovski, 2012; Kundu et al., 2016; Martin & Saller, 1959; Nikolova, Toneva, & Georgiev, 2017; Soni & Khatri, 2016), Sub‐Saharan Africans (De Villiers, 1968; Klopper II, 1943; Rightmire, 1972; Wells, 1947), and the Australian Aborigines (Pardoe, 1984). Actually, an investigation by Hanihara et al. (1998) that covered major populations is the most detailed scholarship on DZ to date. He examined 96 skeletal sample groups in Europe, North America, Australia, and Asia. Ultimately, his findings underscored that East Asians have a relatively higher incidence of DZ than any other geographical group. In fact, results from this work show that the prevalence in the main island of Japan is 2.3%. East and Northeast Asia garner respective rates of 2.8% and 2.5%. These prevalence values far exceed that of other populations. Similarly, the sample with the highest prevalence was a Neolithic sample from Lake Baikal in North Asia, reaching a score of 5.3%. However, this trait is less frequent in Europe than it is in the eastern parts of Eurasia, and DZ is sporadically observed in Oceania and America. Nonetheless, one exception to this concept is in Sub‐Saharan Africa, where DZ has the second highest prevalence found in a geographic group besides Asia (Hanihara et al., 1998). After the comparison between main populations worldwide, Hanihara et al. (1998) concluded that the prevalence denotes geographic interregional clinal variation, from nil to 6.5%, most likely related to genetic factors. Likewise, research in primates by Wang and Dechow (2016) found a prevalence of 0.2% in rhesus macaques, 4.3% in orangutans, but nil in chimpanzees and gorillas. These regional variations in human populations and species differences among nonhuman primates suggest a genetic influence in presence of extra sutures in DZ phenotypes.

Namely, sutures are fibrous joints in vertebrate craniofacial skeletons, serving as important loci of craniofacial growth through their interactions with surrounding tissues and structures (Opperman, 2000; Rice, 2008). Biomechanically, sutures are mechanically weak sites in the otherwise rigid skull (Wang, Smith, et al., 2010; Wang et al., 2012; Wang & Dechow, 2016). Therefore, any disturbance can result in either premature closure of sutures or the addition of extra sutures. These actions alter the normal growth and function of the skull and possibly result in an abnormal skull shape as a consequence of adjusting growth direction and function. Hence, one ramification of this process is that sutural morphology should be well patterned to maintain skull morphology according to species identity (Wang & Dechow, 2016). In nonhuman primates and modern humans, skulls with the DZ condition demonstrated significant morphological features such as asymmetry and regional bony strengthening, suggesting changes in midfacial modularity and functional adaptations (Wang & Dechow, 2016).

While research has covered some parts of the world and incorporated the above primates, unknowns about this condition remain. The purpose of this study was to investigate the DZ phenomenon in Holocene human populations from Northern China. Northern China is a crucial part of East Asia, which contains a diverse history of human integration and migration (Black, 1928; Han, 2009; Wang et al., 2019; Wu & Poirier, 1995; Zhang, 1981; Zhu, 1993, 2014). In this study, the prevalence of DZ and the impact of supernumerary sutures on craniofacial morphology were examined at the macro‐level, particularly for aspects including facial asymmetry, and at the micro‐level for features such as the distribution of cortical bone thickness. This is the first systematic investigation of its kind on skeletal collections of ancient human populations living in Northern China, ranging from the Neolithic Age to recent dynasties. The results will not only enrich our knowledge of DZ cases in terms of regional variations and population relationships, but also allow for better understanding of skull developmental plasticity and patterning.

## 2 METHODS

**Figure 1:**
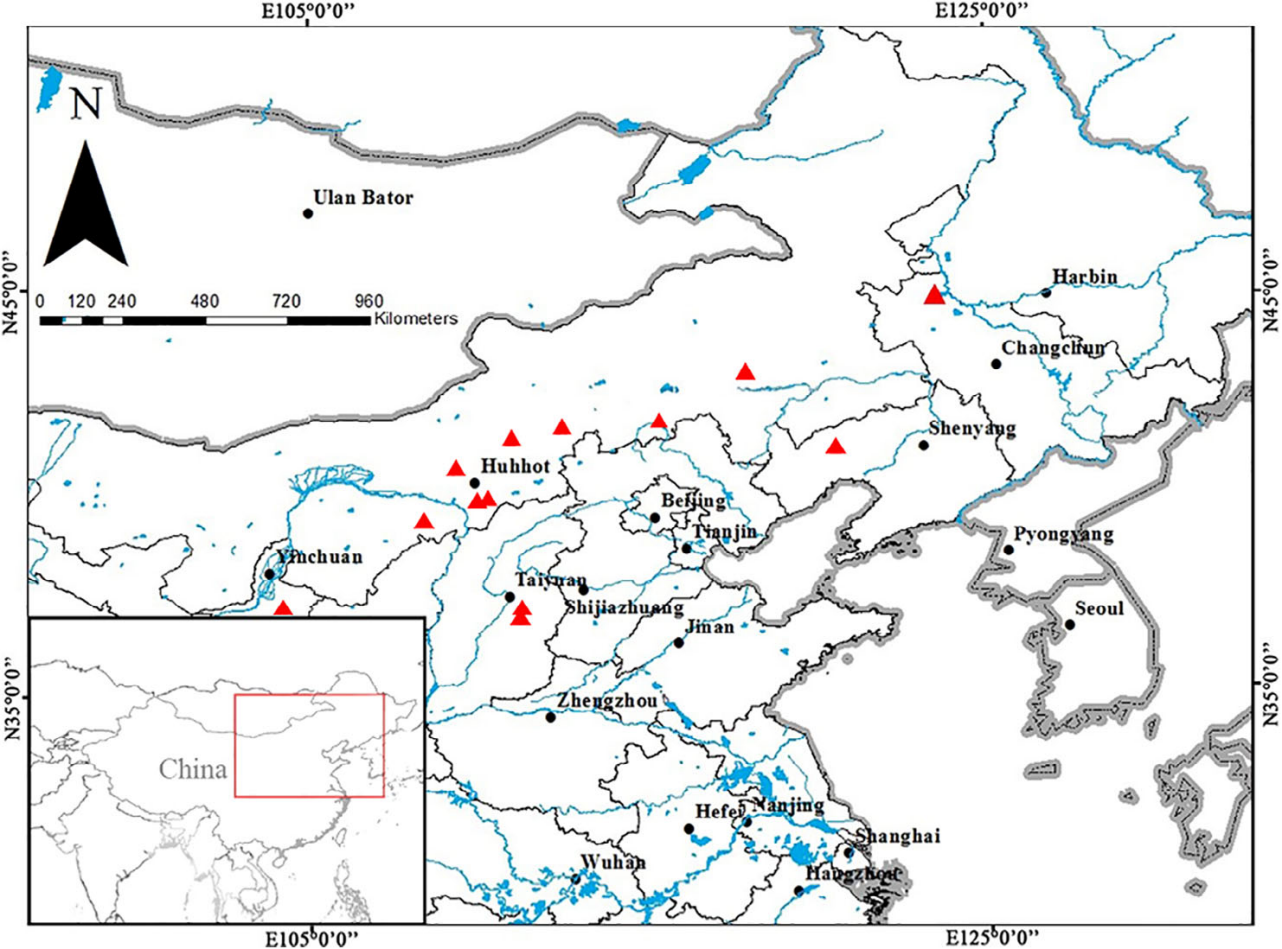
Locations of the investigated populations with DZ samples. Red triangular labels represent the sites where DZ samples were observed

Human skeletons from prehistoric and historic periods stored at the Research Center for Chinese Frontier Archeology at Jilin University were examined for signs of bilateral or unilateral DZ. These collections consisted of various human populations from 49 archeological sites dispersed in Northern China, spanning across the Neolithic period to recent dynasties (5000 to 300 years BP) (Appendix 1, Figure 1). In addition to the two European derived populations from Xinjiang in western China, all other ancient populations involved were Asian with differing archeological cultures. During life, the subsistence patterns of these groups were especially distinct. The Neolithic populations were semi‐agricultural or followed a hunter‐gatherer pattern, while people of the Bronze Age used agricultural and nomadic patterns. In contrast, populations of the Iron Age and the later historical dynasties maintained a settled agricultural pattern. Through their differences, these groups basically represented the main regional subdivisions of the Asian population in northern China. These skulls from this area were also previously analyzed, then generally classified into four different morphological and geographical types (Zhu, 1993, 2002; Zhang, 2010). These types included the East Asian regional group, North Asian regional group, Northeast Asian regional group, and Western East Asian regional group. Moreover, anthropological evidence has demonstrated the diversity of these populations in ancient North China. For example, the East Asian regional group lived in central and eastern China and the North Asian regional group lived in northern China and the Mongolia plateau. Correspondingly, the Northeast Asian regional group resided in northeastern China, whereas the Western East Asian regional group lived in western China (Zhang, 2010; Zhu, 2014). However, it must be noted that these divisions were set and used for the purpose of description only (Wang et al., 2019).

Beyond geographical and cultural classifications, a total of 1145 skulls were visually examined to detect complete division of the zygomatic bone conditions, while partial supernumerary sutures were excluded. On this account, all supernumerary sutures on the identified DZ specimens were further differentiated from specimens with unhealed trauma fractures or other external force fractures. The identified specimens were also examined using a K9500 CBCT scanner (Kodak Dental Systems, Carestream Health Inc. Rochester, New York) within parameters of 80 kV; 80 mA; slice thickness of 0.3 mm in increments of 0.2 mm. Furthermore, prevalence pertaining to the complete horizontal division of the zygoma by the individual and side were calculated separately.

**Figure 2:**
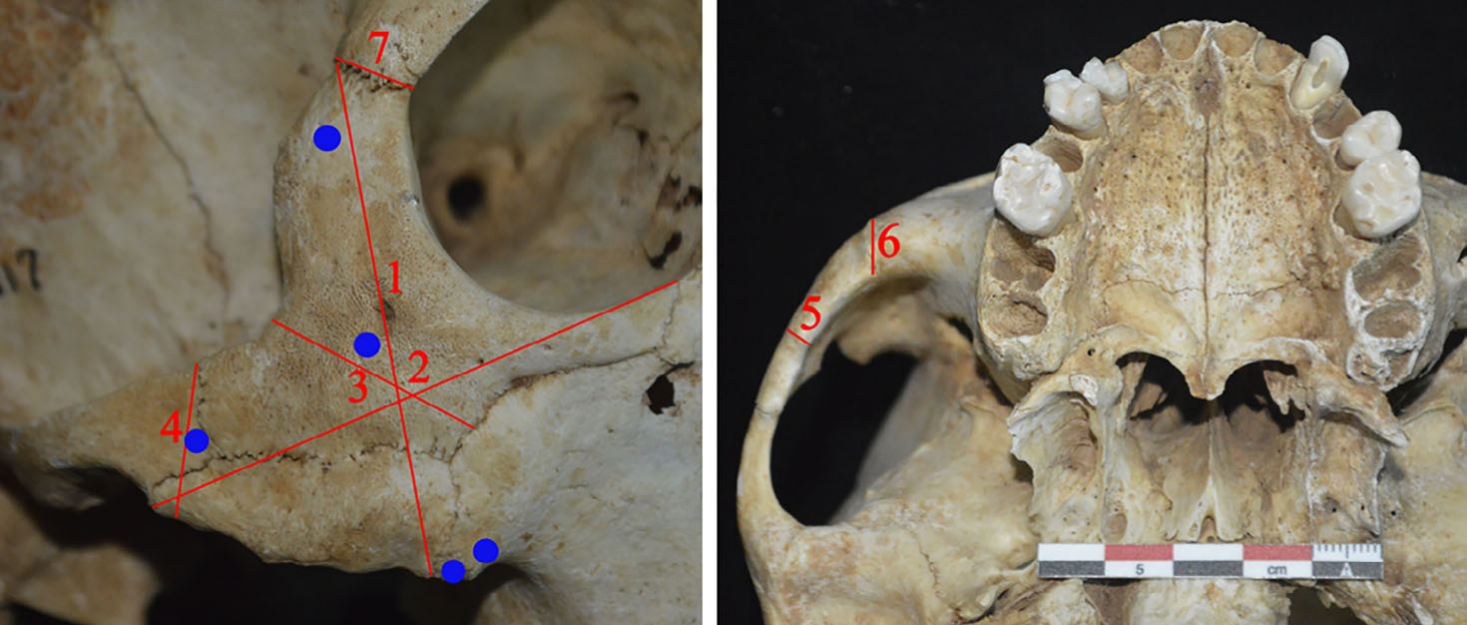
Measurement landmarks on the zygoma. Numbers 1 to 7 in red are: 1: zygoma height, 2: zygoma breadth, 3: zygomatic body minimum breadth, 4: zygomatic arch height, 5: zygomatic arch thickness, 6: zygomatic process of the maxilla thickness, and 7: zygomatic process of the frontal breadth. Five blue points show the locations where the cortical bone thicknesses were measured

The morphology of the craniofacial bones was also recorded by examining the size, shape, and special features of the zygomatic bone, including its height, breadth, area, and symmetry. Specifically, in order to quantify the morphological impacts incurred by the DZ phenomenon, we selected and designed several cranial measurements based on zygoma shape, incorporating upper facial height (n‐pr), height and breadth of the zygoma, minimum breadth of the zygomatic body, the height and thickness of the zygomatic arch, thickness regarding the zygomatic process of the maxilla, and the breadth of the zygomatic process of the frontal bone (Figure 2). The measurements were taken on both sides and different sets of comparisons were designed. By way of example, side‐based comparisons were carried out in unilateral DZ specimens to examine the asymmetry between the DZ side and the normal side by calculating relevant size ratios. Likewise, in bilateral test subjects, the averages of measurements on both sides were calculated. Meanwhile, 42 adult individuals with normal zygoma from the archeological sites were chosen as a control group, with the sex and age numbers of the selected sample similar to that of the DZ samples. In addition, CT images were used to assess facial asymmetry patterns in skulls with unilateral DZ conditions by measuring the thickness of the cortical bone. Specifically, five points located on the body of the zygoma were selected, including the zygomatic process of the maxilla, the frontal zygomatic process, and the zygomatic arch (Figure 2). Correspondingly, determination of cortical bone thicknesses was performed using Mimics Research 16.0 software (Materialise Inc., Leuven, Belgium) to create measurements from virtual CT reconstructions (which were derived from the bone scans). It is also important to mention that all measurements were conducted by the same observer (QZ) three times and average values were recorded.

Data were then analyzed using a statistical analysis program named GraphPad 6.0 for Windows (GraphPad Software Inc., La Jolla, California). Descriptive statistics and Student *t* tests were used to summarize and assess facial skeletal asymmetry. In addition, nonparametric Chi‐square row by column tests for independence were used to assess group differences and their association with facial morphologies. The significance level was set at *α* = 0.05.

## 3 RESULTS

### 3.1 The prevalence of divided zygomas in ancient Chinese populations

Fifteen adult individuals out of 1145 skulls were identified with the DZ phenotype, accompanied by an overall prevalence of 1.3%. Moreover, within the assigned classifications, the prevalence was 0.8% (6/777) in the East Asian regional group, 2.2% (12/546) in the North Asian regional group, and 2.7% (2/75) in the Northeast Asian regional group. In contrast, no individual was identified in the Western East Asian regional group (Tables 1 and 2). Although the sample sizes in each category differ, a deeper examination revealed that the combined North and Northeast Asian regional group had a significantly higher DZ frequency than other groups (Chi‐Square test: *X* ^2^ = 6.475, df = 2, *P* = .0393). There were no sex differences (seven males, eight females) or side differences in terms of the prevalence, which is consistent with previous findings (Hauser & De Stefano, 1989). Furthermore, nine individuals had bilateral DZ, while others were identified as unilateral. In the unilateral cases, three specimens presented signs on the right side and another three on the left side. Those individuals with DZ came from 13 different populations located throughout Northern China, including Inner Mongolia, Jilin, Liaoning, Ningxia, and Shanxi Provinces (Figure 1), and who lived between 5000 and 300 years BP.

**Table 1:** The description of the specimens with divided zygoma

No.	Sample	Period	Sample size	Sex of DZ specimen	Age of DZ specimen	DZ location	Regional type
1	Zhukaigou M1064	3000‐1600 BCE	22	F	30±	Bilateral	East Asian region
2	Baiyan H2005	2000‐1046 BCE	10	M	40±	Bilateral	East Asian region
3	Xindianzi M33:1	600 BCE	25	F	25‐30	Bilateral	North Asian region
4	Jinggouzi M46‐B	500 BCE	27	M	22±	Right	North Asian region
5	Da'an M74C	475 BCE‐220 CE	75	F	35±	Bilateral	North Asian region/Northeast Asian region
6	Da'an M30	475 BCE‐220 CE		M	20‐25	Bilateral	
7	Dongdajing M4:2	8‐220 CE	11	F	15‐16	Left	North Asian region
8	Qilangshan M18	220‐420 CE	4	F	25‐30	Bilateral	North Asian region
9	Tuchengzi M170	220‐420 CE	103	F	25‐30	Bilateral	North Asian region
10	Lamadong M217	300‐420 CE	155	M	24‐35	Right	East Asian region/North Asian region
11	Lamadong M371	300‐420 CE		M	35±	Left	East Asian region/North Asian region
12	Sanmianjing M6	1271‐1368 CE	6	M	20±	Right	North Asian region
13	Zhanzishan M68	1271‐1368 CE	29	M	25±	Bilateral	East Asian region/North Asian region
14	Kaicheng M65	1271‐1368 CE	12	F	35‐50	Left	North Asian region
15	Yuci Mf5‐1	1368‐1800 CE	111	F	30‐35	Bilateral	East Asian region

**Table 2:** The prevalence of divided zygoma among different Asian regional subgroups

Sample name	Right (%)	Left (%)	By individual (%)	By side (%)
East Asian regional group	0.64 (5/777)	0.64 (5/777)	0.77 (6/777)	0.64 (10/1554)
North Asian regional group	1.65 (9/546)	1.65 (9/546)	2.20 (12/546)	1.65 (18/1092)
Northeast Asian regional group	2.67 (2/75)	2.67 (2/75)	2.67 (2/75)	2.67 (4/150)
Western East Asian region group	0.00 (0/60)	0.00 (0/60)	0.00 (0/60)	0.00 (0/120)
Total	1.05 (12/1145)	1.05 (12/1145)	1.31 (15/1145)	1.05 (24/2290)

*Note*: six archeological sites contained two regional groups which they were included in both groups. Chi‐square test: Four groups, *X* ^2^ = 6.582, df = 3, *P* = .0865; three groups with North and Northeast combined *X* ^2^ = 6.475, df = 2, *P* = .0393. The combined North and Northeast Asian regional group had significantly higher prevalence than the other groups.

### 3.2 Side‐based morphological difference in unilateral DZ specimens

In an additional exploration of our results, the measurements on both sides in unilateral DZ skulls displayed a significant asymmetry between the DZ side and the normal side (Table 3). Overall, total zygoma height, breadth, and area on the DZ side were larger than that of the normal side, whereas the minimum breadth of the zygomatic body was smaller on the DZ side than on the normal side. In the inferior part of the skull, the height, thickness, and area of zygomatic arch along with the thickness of the zygomatic process of the maxilla were both larger than on the normal side. Conversely, on the superior part, the breadth of the frontal zygomatic process was relatively smaller than on the normal side.

**Table 3:** Linear measurements of skulls with unilateral DZ

Specimens with unilateral DZ	Jinggouzi	Sanmianjing	Lamadong	Dongdajing	Lamadong
	M46:B	M6	M217	M4:2	M371
DZ side	Right	Right	Right	Left	Left
Upper facial height	73.45	82.30	74.92	74.92	69.21
Zygoma height right	**59.04**	**59.74**	**49.66**	48.76	43.55
Zygoma breadth right	**57.81**	**59.23**	**57.46**	50.11	43.96
Zygoma height left	54.03	53.99	47.43	**56.27**	**48.03**
Zygoma breadth left	57.72	56.09	/	**50.56**	**/**
Area of zygoma^1^ right	**3413.10**	**3538.40**	**2853.46**	2443.36	1914.46
Area of zygoma left	3118.61	3028.30	/	**2845.01**	**/**
Zygomatic body minimum breadth right	**21.82**	**24.32**	**22.92**	24.54	19.45
Zygomatic body minimum breadth left	22.64	25.71	/	**23.2**	**14.35**
Zygomatic arch height right	**17.63**	**18.50**	**16.75**	14.59	9.58
Zygomatic arch thickness right	**5.32**	**5.83**	**4.46**	4.75	4.12
Zygomatic arch height left	15.04	15.97	/	**22.36**	**/**
Zygomatic arch thickness left	4.77	5.49	/	**4.83**	**/**
Area of zygomatic arch^2^ right	**93.79**	**107.86**	**74.71**	69.30	39.47
Area of zygomatic arch left	71.74	87.68	/	**108.00**	**/**
Zygomatic process of the maxilla thickness right	**10.03**	**12.90**	**8.99**	8.7	6.66
Zygomatic process of the maxilla thickness left	9.50	12.22	/	**9.28**	**7.58**
Zygomatic process of the frontal breadth right	**8.15**	**7.48**	**8.71**	9.06	7.93
Zygomatic process of the frontal breadth left	8.71	7.73	10.66	**8.35**	**/**

*Note*: 1: the area of the zygoma is zygoma height times zygoma breadth; 2: the area of the zygomatic arch is zygomatic arch height times zygomatic arch thickness. DZ side was highlighted in bold. Unit: mm. “/” indicates that the measurement item is unavailable on the specimen.

**Figure 3:**
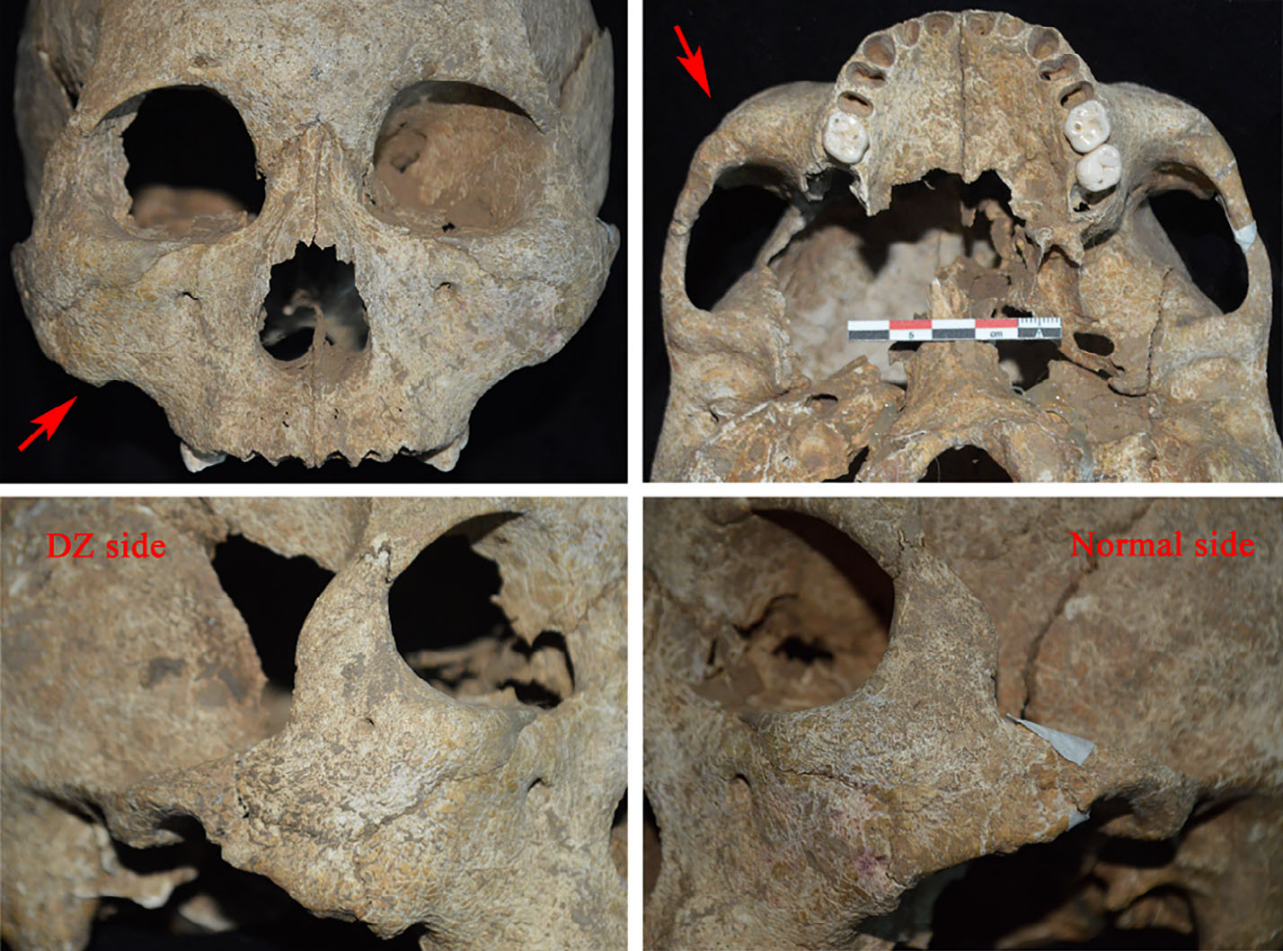
Illustration of a unilateral DZ skull (Jinggouzi M46B) from the anterior, inferior, and side orientations. Remarkable facial morphological asymmetries presented between the DZ side and the normal side

**Figure 4:**
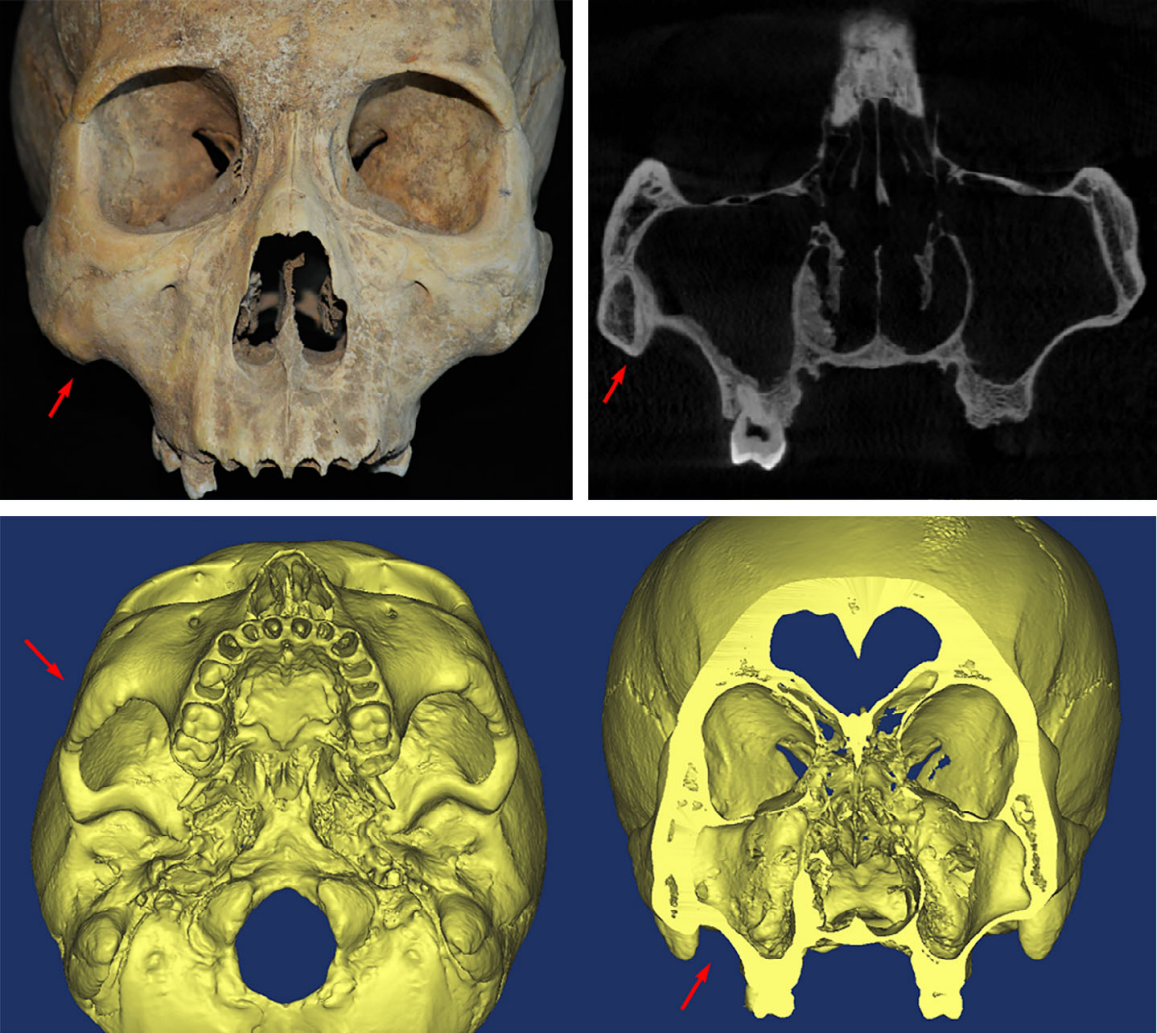
Unilateral DZ conditions illustrated on macroscopic and CT images (Sanmianjing M6). The red arrows indicate the thickening of cortical bone on the DZ side

Furthermore, these unilateral DZ skulls demonstrated a side‐based asymmetry larger than in normal subjects (Table 4; Figure 3). In general, the area of the zygoma on the DZ side was larger than on the normal side, with an average ratio of 1.14. Accordingly, in the inferior part of the zygoma, the area of the zygomatic arch and the zygomatic process of the maxilla thickness were larger on the DZ side with average ratios of 1.36 and 1.13, respectively. In the superior part of the zygoma, the minimum breadth of the zygomatic body and the breadth of the frontal zygomatic process were smaller on the DZ side, with respective average ratios of 0.90 and 0.91. Finally, CT images showed that the cortical bone thickness measurements were larger on the DZ sides than on the corresponding normal sides (Table 5; Figure 4).

**Table 4:** Ratios of side‐based differences on unilateral DZ skulls

	Ratio of side‐based difference	Area of zygoma	Zygomatic body minimum breadth	Area of zygomatic arch	Zygomatic process of the maxilla thickness	Zygomatic process of the frontal breadth
Jinggouzi M46:B	DZ/Left	1.09	0.96	1.31	1.23	0.94
Lamadong M371	DZ/Right	/	0.74	/	1.14	/
Lamadong M217	DZ/Left	/	/	/	/	0.82
Sanmianjing M6	DZ/Left	1.17	0.95	1.23	1.06	0.97
Dongdajing M4:2	DZ/right	1.16	0.95	1.56	1.07	0.92
DZ mean (N = 5)	DZ/normal Mean ± SD	1.14 ± 0.04	0.90 ± 0.11	1.36 ± 0.17	1.13 ± 0.79	0.91 ± 0.07
Normal mean (N = 42)	Right/left Mean ± SD	1.00 ± 0.05	1.01 ± 0.04	1.03 ± 0.14	1.01 ± 0.10	0.98 ± 0.07
*P* value		<.001	.128	<.001	.025	.084

*Note*: In unilateral DZ specimens, the values of the DZ side were used as the numerator, while the values of the normal side were used as the denominator.

**Table 5:** Comparison of cortical bone thickness between DZ and normal sides on unilateral DZ samples

	Most lateral point on the frontal process of the zygomatic bone		Most anterior‐lateral point on zygoma body		Midpoint of vertical from the superior point of the temporozygomatic suture		Most inferior point on the zygomatic process of the maxilla		Most anterior point on the zygomatic process of the maxilla		Overall mean thickness of the five points		*P* value^2^
	DZ	Normal	DZ	Normal	DZ	Normal	DZ	Normal	DZ	Normal	DZ	Normal	
Sanmianjing M6	2.36	1.98	1.83	1.03	1.86	1.39	2.06	1.75	2.06	1.45	2.03	1.52	0.004
Jinggouzi M46:B	2.16	1.65	1.87	1.48	1.63	1.07	1.95	1.27	1.65	1.32	1.85	1.36	0.001
Lamadong M371	2.11	1.79	1.75	1.39	1.57	1.09	1.98	1.57	2.32	1.67	1.95	1.50	0.002
Lamadong M217	2.01	1.32	1.86	1.19	1.80	1.39	1.95	1.53	1.92	1.51	1.91	1.39	0.001
Mean	2.16	1.69	1.83	1.27	1.72	1.24	1.99	1.53	1.99	1.49	1.94	1.44	<0.001
*P* value^1^	.010		.014		.001		.010		.007		<.001		

*Note*: 1: Student *t* tests between sides among four individuals; 2: Student *t* tests between sides among five points.

### 3.3 Differences between bilateral skulls and normal skulls

**Figure 5:**
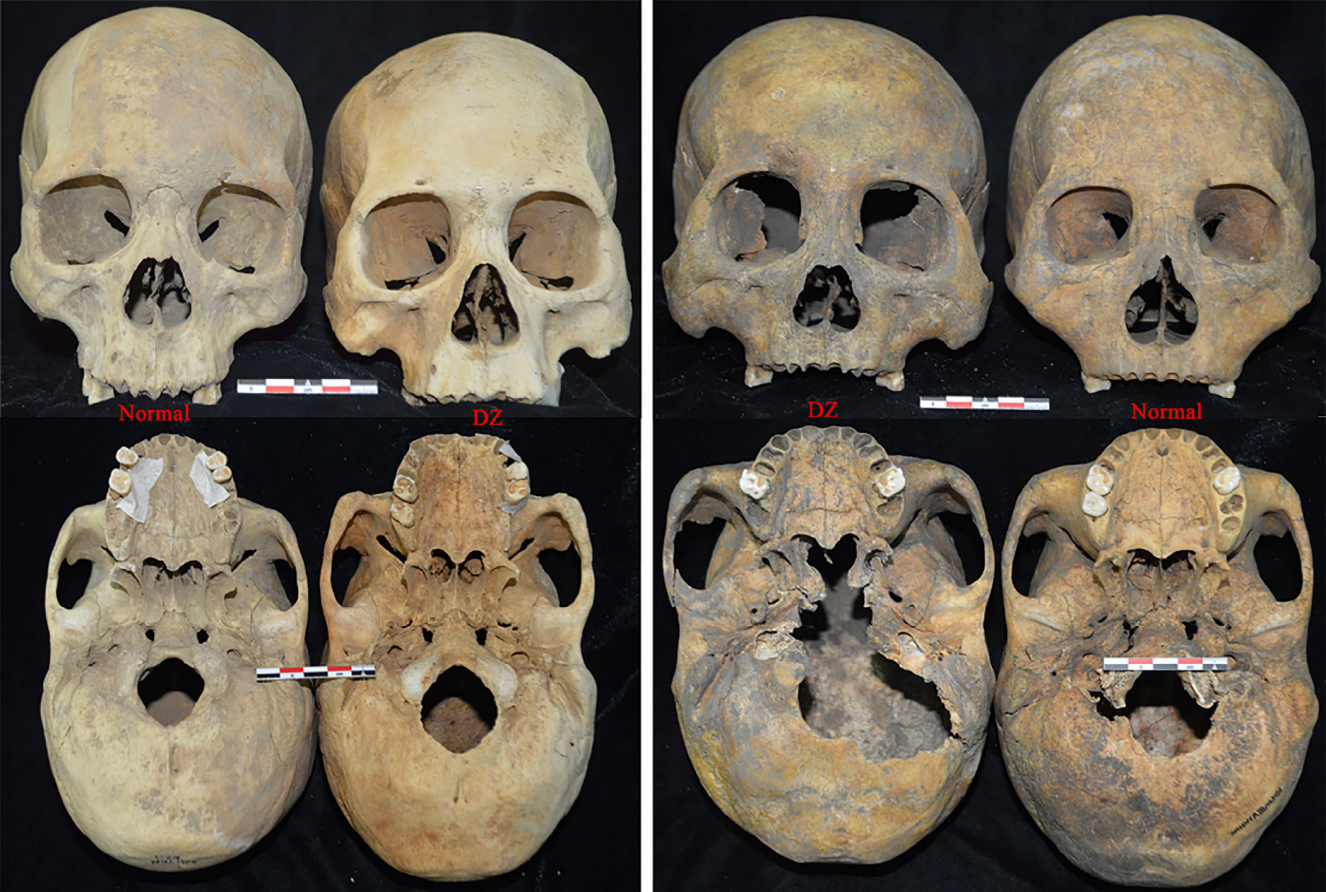
Illustrations of the bilateral DZ skull (Yuci Mf5‐1 and Yuci Mb7‐1 on the left, Da'an M74C and Da'an M39A are on the right). Remarkable facial morphological differences are shown between the DZ skulls and the normal skulls along the inferior border of the maxillary and zygomatic bones

**Figure 6:**
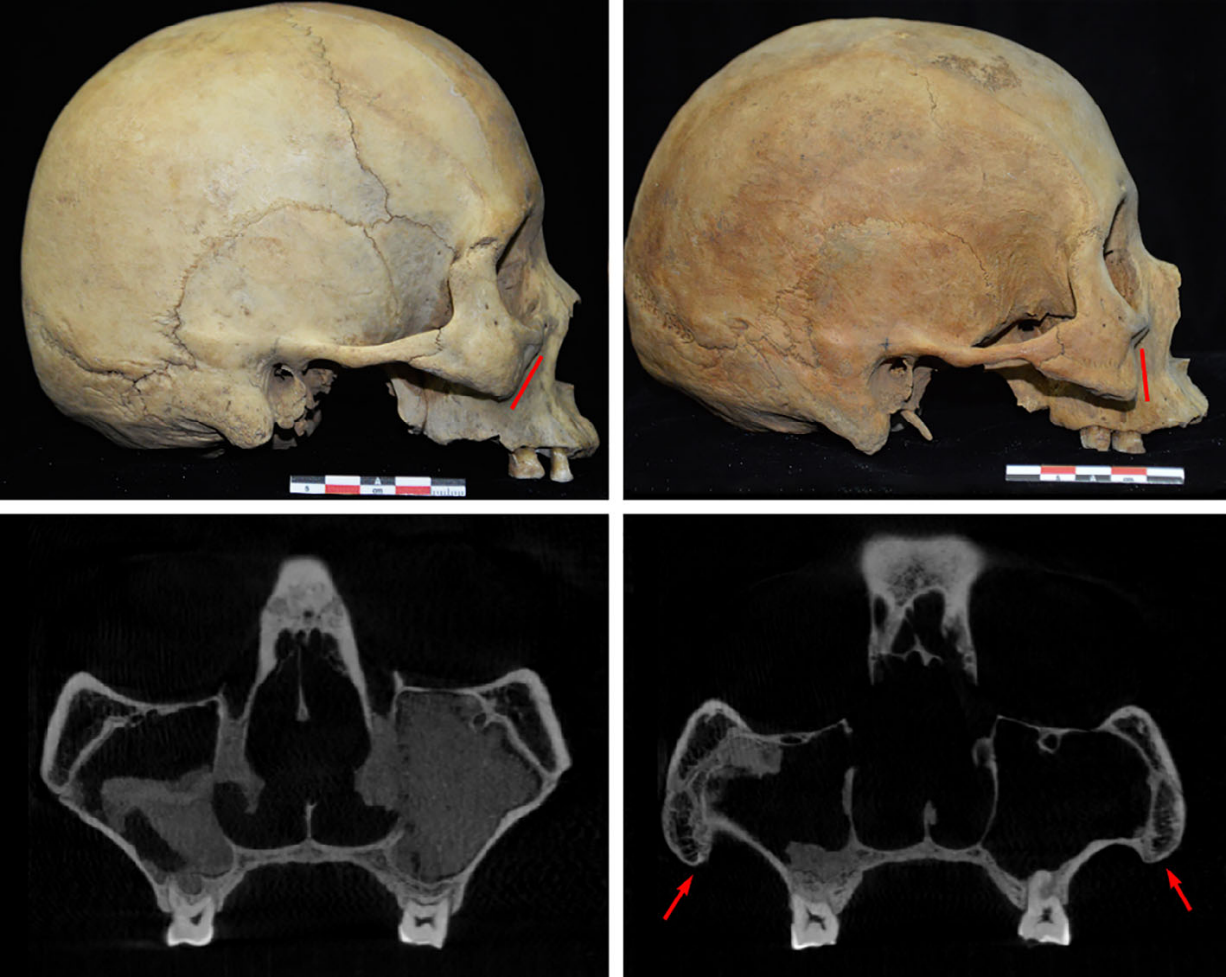
Bilateral DZ conditions illustrated on macroscopic and CT images (Yuci Mf5‐1 and Yuci Mb7‐1 on the top, Da'an M74C and Da'an M39A below). The red lines indicate the normal shape and “wedge‐like process” of the maxilla with the laterally extended and curved inferior borders in the DZ skull, while the red arrows show morphological differences associated with structural strengthening on the inferior borders in the DZ skull

The size and shape of the zygomatic bones were also different between bilateral DZ skulls and normal skulls (Table 6; Figure 5). In bilateral DZ specimens, not only was the minimum breadth of the zygomatic body smaller in typical humans than on the DZ skull, the ratio of zygoma height to zygoma breadth was larger on the DZ skull as well. Meanwhile, in a comparison of zygoma size to overall facial size, the zygoma height was larger relative to the upper facial height in DZ skulls, while the zygoma breadth showed no significant differences relative to midfacial breadth, indicating that the zygoma in bilateral DZ skulls was more slender relative to the whole facial skeleton than in the normal skulls (Table 6). Simultaneously, the lower division of the zygoma and the adjacent bones demonstrated structural changes indicating increased strength. This finding was especially true for the maxilla, as both sides had a “wedge‐like process” that extended laterally with curved inferior borders, resulting in remarkably prominent muscle attachment markings (Figure 6). The area of zygomatic arch was also much larger on DZ skulls, suggesting greater strength. In addition, differences between the superior and inferior divisions in bilateral DZ skulls were bigger than in normal skulls. Moreover, compared to normal skulls, the ratio of the zygomatic arch height to the breadth of the frontal zygomatic process was much larger, generating a consistency with observations that the bilateral DZ specimens have relatively small zygomatic processes of the frontal bone and a larger zygomatic arch size The middle facial breadth and the upper facial height showed no significant differences (Table 6).

**Table 6:** Comparisons between bilateral DZ and normal skulls on zygomatic morphology

		Zygomatic arch height/zygomatic process of the frontal breadth	Zygoma height/zygoma breadth	Zygoma height/upper facial height	Zygoma breadth/middle facial breadth	Zygomatic body minimum breadth	Area of zygomatic arch
Male	Da'an M30	2.27	1.11	0.83	0.48	21.97	67.92
	Zhenzishan M68	2.34	1.06	0.82	0.56	20.21	91.10
	Baiyan H2005	1.78	1.01	0.81	0.54	23.30	89.19
	DZ mean (N = 3) Mean ± SD	2.13 ± 0.31	1.06 ± 0.05	0.82 ± 0.01	0.52 ± 0.04	21.83 ± 1.55	82.74 ± 12.87
	Normal mean (N = 17) Mean ± SD	1.66 ± 0.29	0.88 ± 0.05	0.69 ± 0.03	0.55 ± 0.04	27.15 ± 2.16	68.67 ± 17.86
	*P* value	.020	<.001	<.001	.167	.001	.212
Female	Zhukaigou M1064	2.20	1.05	0.73	0.52	24.58	86.25
	Xindianzi M33:1	2.16	1.06	0.78	0.53	20.48	62.19
	Da'an M74C	2.34	1.15	0.89	0.51	23.78	80.20
	Qilangshan M18	2.09	1.00	0.78	0.54	21.81	94.37
	Yuci Mf5‐1	2.04	1.22	0.82	0.50	19.62	97.32
	DZ mean (N = 5) Mean ± SD	2.16 ± 0.12	1.10 ± 0.09	0.80 ± 0.06	0.52 ± 0.02	22.05 ± 2.11	84.07 ± 13.97
	Normal mean (N = 25) Mean ± SD	1.65 ± 0.29	0.89 ± 0.08	0.67 ± 0.03	0.53 ± 0.06	25.12 ± 2.86	66.90 ± 16.88
	*P* value	.001	<.001	.007	.671	.032	.043

## 4 DISCUSSION

### 4.1 The prevalence of DZ in different populations

This is the first systematic investigation of the DZ phenomenon in Holocene human populations from Northern China. Forty‐nine ancient populations that lived in the Northern China area for over 5000 years represented the main population types studied. Except for the two European derived groups in western China, the specimens consisted of relatively isolated Asian populations with different cultures and subsistence patterns. The frequency of DZ in the whole ancient Chinese population was found to be 1.3%, with a higher incidence of 2.3% in North Asian and Northeast Asian regional groups combined, demonstrating a difference of prevalence among separate regional groups. Otherwise, there were no sex or side differences. In larger context, a comparison with other main populations worldwide asserts that the incidence of this phenomenon in the East Asian populations is relatively higher, indicating that there could be some genetic factors behind the occurrence of DZs. However, in further regard to the Chinese population, very limited investigations have been conducted on either modern Northern or Southern Chinese (Table 7). The populations from Yingchuan (Li, 1985a), Gansu (Li, 1985b), Shaanxi (Li, 1985b), and Qingdao (Ding, 1961), which represented the Northern Chinese, have a respective prevalence of 2.3%, 3.6%, and 1.3%, while the populations from Nanjing (Gong & Du, 1965), Jiangxi (Hu et al., 1985), and Kunming (Yang, 1987), which represented the Southern Chinese, have their own respective prevalence rates at 0.5%, 0.7%, and 0.2%. These prevalence values show a decline from the Northern to the Southern populations, suggesting clinal variation. For example, there is a high DZ prevalence among North Asian regional populations, who were characterized by flat and broad faces, suggesting a morphological or genetic link to this phenomenon. Generally, Asians are characterized by high cheek bones with flat faces. This point is especially reinforced among North Asian and Northeast Asian regional populations. In these groups, there are very flat faces in the transverse midfacial plane, along with lateral and forward positioned zygomas (Baba & Narasaki, 1991; Chen et al., 2011; Hamilton, 2008; Hanihara, 2000; Hanihara, Ishida, & Dodo, 2003; Oettlé et al., 2017). A few Sub‐Saharan Africans also present similar characteristics with flat faces and projecting zygomas (Hanihara, 2000). Intriguingly, the prevalence of DZ attained a higher rate in both the East Asian and South African populations. Indeed, this particularly high prevalence was among the populations with flat faces and projecting zygomas. Thus, it is hypothesized that DZs develop more often in individuals with broad facial morphologies. In context, broad facial morphology has been linked to increased anterior teeth loading stress and efficiency (Wang et al., 2019; Wang, Wright, Smith, Chalk, & Byron, 2010). Hence, the presence of supernumerary sutures in the midface under a high stress environment will have a negative impact, which in turn demands a functional and morphological adaptation to alleviate the situation. This reaction may represent an example of developmental plasticity in the craniofacial skeleton (Wang & Dechow, 2016).

**Table 7:** The prevalence of DZ in various populations

Populations		By individual (%)	By side (%)	Resource
Yingchuan	Northern Chinese	3.0 (6/200)	2.25 (9/400)	Li, 1985a
Gansu, Shaanxi		3.92 (13/332)	3.61 (24/664)	Li, 1985b
Qingdao		/	1.28 (21/1638)	Ding, 1961
Nanjing	Southern Chinese	0.59 (6/1018)	0.49 (10/2036)	Gong & Du, 1965
Jiangxi		0.88 (7/800)	0.69 (11/1600)	Hu, Li, & Zhen, 1985
Kunming		/	0.24 (4/1666)	Yang, 1987
East Asia		2.79 (8/287)	1.98 (12/607)	Hanihara et al., 1998
Northeast Asia		2.53 (13/514)	1.36 (15/1101)	Hanihara et al., 1998
Southeast Asia		0.99 (11/1115)	0.65 (15/2320)	Hanihara et al., 1998
Subsaharan Africa		0.81 (6/743)	0.67 (11/1648)	Hanihara et al., 1998
North Africa		0.75 (5/669)	0.49 (7/1434)	Hanihara et al., 1998
Indian subcontinent		0.57 (4/696)	0.49 (7/1438)	Hanihara et al., 1998
Europe		0.45 (6/1336)	0.32 (9/2836)	Hanihara et al., 1998
Oceania		0.10 (2/2015)	0.07 (3/4354)	Hanihara et al., 1998
West‐central Asia		0.00 (0/312)	0.14 (1/711)	Hanihara et al., 1998
Arctic group		0.00 (0/376)	0.12 (1/809)	Hanihara et al., 1998
American Indians		0.00 (0/490)	0.00 (0/1120)	Hanihara et al., 1998

Developmental plasticity is defined as “the ability of an organism to react to an internal or external environmental input with a change in form, state, movement, or rate of activity” (West‐Eberhard, 2003). To explain, selection can act on phenotypes expressed in developmentally plastic systems in response to environmental factors, thereby linking developmental plasticity to adaptive evolutionary change (West‐Eberhard, 2005). In the case of the highly integrated aspects within craniofacial skeletons, plasticity in the midface and surrounding areas is fundamental to their ability to respond independently or collectively to environmental conditions and ultimately their ability to respond adaptively to selection. Hence, although this study has identified the rates of DZ itself in China, developmental plasticity in populations with broad faces and the tendency to have a high incidence of DZ warrants more investigation. The skeletal collections of Neolithic and Dynastic population in Northern China have a chronological depth that shows in‐situ evolution, and thus provides a rare opportunity for the study of the evolution and adaptation of craniofacial morphology in modern humans. Further insights into this rare DZ condition would deepen our understanding of craniofacial form, adaptation, developmental plasticity, and evolution.

### 4.2 The occurrences and mechanisms of divided zygoma

One explanation for the rarity of this condition is that it could be a random mutation related to the developmental failure of sutural fusion. The mechanisms that cause the supernumerary sutures (such as those displayed in DZs) remain unclear. Typically, the horizontal supernumerary sutures on the DZ samples were located on the relatively fixed inferior part of the zygoma ‐ a term used when dividing the zygoma into portions, with a larger superior part and a smaller inferior part. In the case of the unilateral DZ samples, the incomplete horizontally divided zygomatic suture can also be observed at the same location on the other side (Table 8). These phenomena suggest that the occurrence of the supernumerary sutures should be related to the number of ossification centers in the ontogeny and development of the bone (Bhargava et al., 1960; De Villiers, 1968; Jeyasingh et al., 1982). Generally, it is thought that the human zygoma has only one ossification center, which appears in a fetus at eight weeks (Cunningham, 1947; Grant, 1952; Mall, 1906; Morris & Schaeffer, 1953; Woo, 1956). However, more ossification centers develop in some circumstances. Buchanan's Manual of Anatomy emphasizes that three ossification centers of the anterior, posterior, and inferior parts of the zygoma fuse to form the mature bone. Accordingly, the DZ will occur if these three ossification centers fail to fuse, resulting in *zygomaticum bipartium* or more divisions (Jones, 1946).

**Table 8:** The occurrence of incomplete sutures on the normal side in the unilateral DZ skulls

No.	Site	Sex	Age	Location	Incomplete sutures
1	Jinggouzi M46‐B	M	22±	Right	Posterior
2	Dongdajing M4:2	F	15‐16	Left	Posterior
3	Sanmianjing M6	M	20±	Right	Posterior
4	Kaicheng M65	F	35‐50	Left	Anterior
5	Lamadong M217	M	24‐35	Right	Anterior
6	Lamadong M371	M	35±	Left	N/A

Also, from the perspective of calvarial embryogenesis, wormian bones and associated supernumerary sutures vary in different parts of the cranium. These conditions are usually associated with areas such as the lambda and bregma, as they develop fontanelles during the early stages of skull ontogeny (Anton, Jaslow, & Swartz, 1992). Moreover, both the zygoma and cranium are derived from intramembranous bone, suggesting similarities in developmental pathways. DZ have been observed on some basal tetrapods (Stegocephalia) as well, suggesting that these pathways and their perturbations are ancient in vertebrate cranial development and evolution (Gong & Du, 1965).

### 4.3 The effect of DZ on craniofacial morphology and the adaptability of morphological mechanisms

As observed by Wang and Dechow (2016) in rhesus monkeys, there were remarkable side‐based differences in unilateral DZ skulls with facial asymmetry due to the differences in the size of the zygoma and subtle differences in adjacent bones, including the maxillary, temporal, and frontal bones. For instance, the superior division of the zygoma was more gracile compared to the normal side, while the inferior division was more robust. The same facts were true for bilateral DZ skulls compared to normal skulls. Thus, the DZ morphology consequently disturbs developmental modularity and the normal inferosuperior buttressing of biting and masticatory loads from the maxilla to the frontal, leading to facial asymmetries, regional strengthening of the cortical bone, and changes in patterns of muscle attachment (Wang & Dechow, 2016).

Morphological adaptions within and surrounding the DZ (including the cortical thickness asymmetries) led us to ponder the correlations between morphology and functional adaptation. Functional adaptations of bone can be a response to changes in mechanical stress. For example, as the outer layer of a bone organ, cortical bone grows and remodels in response to changes in mechanical loading and increased bone strain (Baab, Copes, Ward, Wells, & Grine, 2018; Lanyon, Goodship, Pye, & Macfie, 1982; Robling, Hinant, Burr, & Turner, 2002; Warden et al., 2005). Previous studies have shown in detail that the presence or absence of muscle and the degree of muscle attachment have an impact on cortical bone properties. The bone underlying the muscle‐bearing sites may adapt to the local “strain microenvironment” by increasing maximum stiffness (Peterson & Dechow, 2003). In the DZ condition, the morphology of the zygoma likely adapt to loading changes caused by the presence of a supernumerary suture such that some local structures in maxillary bone and zygomatic arch are strengthened to allow sufficient muscle attachment and muscle force for orofacial activities. Accordingly, the cortical bone on the DZ side thickens in coordination with morphological shape change in response to function.

## 5 CONCLUSION

DZ is a rare yet significant trait that allows the study of ontogenetic variations in the supernumerary sutures of skulls and their biomechanical effects. In the present study, crania from Northern China retrieved in archeological excavations were examined to determine the prevalence of the DZ condition in these Holocene populations for the first time. Fifteen adult individuals were identified with types of DZ out of the 1145 skulls examined, giving a prevalence of 1.3% overall, along with a greater prevalence of 2.2% in the North Asian regional group and 2.7% in the Northeast Asian regional group. This pattern of distribution led to the hypothesis that the DZ trait is more frequent in populations characterized by flat and broad faces. Further, our measurements of zygoma morphology and cortical bone thickness indicated remarkable differences between divided and normal zygoma in both macroscopic morphology and microstructure. The superior division of the DZ was normally more slender along with a weaker adjacent frontal bone articulation; while the inferior division of the DZ was normally more robust, including correspondingly stronger temporal and maxillary bones. These adaptations in bone morphology demonstrate that a supernumerary suture within the zygoma may alter the pattern of stress distribution in the midface during function and, therefore, affect both macroscopic and microscopic aspects of facial morphology. Our findings reveal relationships between craniofacial features, geographic location, and substance pattern, which warrants more morphological and ethnogeographic studies toward a better understanding of this interesting feature.

## AUTHOR CONTRIBUTIONS

Q.W., Q.C.Z., H.Y., and Q.Z. designed the study and directed implementation. Q.Z., S.Y., and Q.W. collected the data. Q.Z. and Q.W. analyzed the data and drafted the manuscript. Q.W., P.C.D., H.Y., Q.C.Z. and H.Z. edited the manuscript for intellectual content and provided critical comments on the manuscript.

## Appendix

**Table:**

	Site	Period	Sample size	Location	Regional type
1	Baiyinchanghan	4000 BCE	1	Inner Mongolia	East Asian region
2	Miaozigou	3000 BCE	9	Inner Mongolia	East Asian region
3	Dabagou	3000 BCE	2	Inner Mongolia	East Asian region
4	Zhukaigou	3000‐1600 BCE	22	Inner Mongolia	East Asian region
5	Baiyan	2000‐1046 BCE	10	Shanxi	East Asian region
6	Xicha	2500‐2000 BCE	10	Inner Mongolia	East Asian region
7	Mapai	2200‐2000 BCE	3	Qinghai	East Asian region
8	Dashanqian	2000‐1500 BCE	2	Inner Mongolia	East Asian region
9	Xiaohandi	1600 BCE	13	Qinghai	East Asian region
10	Daihai	1500‐500 BCE	14	Liaoning	East Asian region
11	Longtoushan	1046‐771 BCE	12	Inner Mongolia	East Asian region
12	Lichengxiguan	1046‐771 BCE	4	Shanxi	East Asian region
13	Ximaqing	771 BCE	9	Inner Mongolia	East Asian region
14	Xindianzi	600 BCE	25	Inner Mongolia	North Asian region
15	Jinggouzi	500 BCE	27	Inner Mongolia	North Asian region
16	Bancheng	500 BCE	24	Inner Mongolia	North Asian region/East Asian region
17	Guoxianyaozi	500 BCE	5	Inner Mongolia	North Asian region/East Asian region
18	Shuanggucheng	500‐400 BCE	4	Inner Mongolia	North Asian region/East Asian region
19	Yanghai	500‐400 BCE	49	Xinjiang	Western East Asian region
20	Jilintai	500‐100 BCE	11	Xinjiang	Western East Asian region
21	Da'an	475 BCE‐220 CE	75	Jilin	North Asian region/Northeast Asian region
22	Houchengzui	475‐221 BCE	5	Inner Mongolia	East Asian region
23	Dongtouhao	475‐221 BCE	14	Inner Mongolia	East Asian region
24	Jiangjungou	300‐221 BCE	14	Inner Mongolia	East Asian region
25	Yinniugou	200 BCE	20	Inner Mongolia	East Asian region
26	Zhalainuoer	202 BCE‐220 CE	7	Inner Mongolia	North Asian region/East Asian region
27	Fenghuangshan	202 BCE‐220 CE	3	Inner Mongolia	‐
28	Xitun	202 BCE‐300 CE	130	Beijing	East Asian region
29	Taojiazhai	202 BCE‐300 CE	127	Qinghai	East Asian region
30	Sandaowan	8‐220 CE	10	Inner Mongolia	North Asian region
31	Dongdajing	8‐220 CE	11	Inner Mongolia	North Asian region
32	Bagou	8‐266 CE	4	Inner Mongolia	North Asian region
33	Damaoqi	220‐420 CE	6	Inner Mongolia	North Asian region
34	Qilangshan	220‐420 CE	4	Inner Mongolia	North Asian region
35	Tuchengzi	220‐420 CE	103	Inner Mongolia	North Asian region
36	Lamadong	300‐420 CE	155	Liaoning	East Asian region/North Asian region
37	Danjiadian	300‐420 CE	1	Inner Mongolia	North Asian region
38	Shanzuizi	916‐1125 CE	10	Inner Mongolia	North Asian region
39	Yelvyuzhi	916‐1125 CE	4	Inner Mongolia	North Asian region
40	Dongshan	916‐1125 CE	3	Inner Mongolia	North Asian region
41	Qianhaizi	916‐1125 CE	2	Inner Mongolia	North Asian region
42	Chengbuzi	1115‐1368 CE	17	Inner Mongolia	East Asian region/North Asian region
43	Woniushi	1271‐1368 CE	1	Inner Mongolia	North Asian region
44	Yikeshu	1271‐1368 CE	4	Inner Mongolia	East Asian region
45	Wulagai	1271‐1368 CE	1	Inner Mongolia	North Asian region
46	Sanmianjing	1271‐1368 CE	6	Inner Mongolia	North Asian region
47	Zhenzishan	1271‐1368 CE	29	Inner Mongolia	East Asian region/North Asian region
48	Kaicheng	1271‐1368 CE	12	Ningxia	North Asian region
49	Yuci	1368‐1800 CE	111	Shanxi	East Asian region
Total			1145		
